# Oral Health Workforce in Africa: A Scarce Resource

**DOI:** 10.3390/ijerph20032328

**Published:** 2023-01-28

**Authors:** Jennifer E. Gallagher, Grazielle C. Mattos Savage, Sarah C. Crummey, Wael Sabbah, Benoit Varenne, Yuka Makino

**Affiliations:** 1 Dental Public Health, King’s College London, Faculty of Dentistry, Oral & Craniofacial Sciences, Denmark Hill Campus, London SE5 9RS, UK; 2 Dental Office, WHO Oral Health Programme NCD Department, Division of UHC/Communicable and NCDs, World Health Organization, 20 Avenue Appia, Geneva 1211, Switzerland; 3 Dental Office, Noncommunicable Diseases Team, WHO Regional Office for Africa, Cité Djoué, Brazzaville P.O. Box 06, Congo

**Keywords:** Africa, oral health, health workforce, dental care, low-income population, developing countries

## Abstract

The World Health Organization (WHO) African Region (AFR) has 47 countries. The aim of this research was to review the oral health workforce (OHWF) comprising dentists, dental assistants and therapists, and dental prosthetic technicians in the AFR. OHWF data from a survey of all 47 member states were triangulated with the National Health Workforce Accounts and population data. Descriptive analysis of workforce trends and densities per 10,000 population from 2000 to 2019 was performed, and perceived workforce challenges/possible solutions were suggested. Linear regression modelling used the Human Development Index (HDI), years of schooling, dental schools, and levels of urbanization as predictors of dentist density. Despite a growth of 63.6% since 2010, the current workforce density of dentists (per 10,000 population) in the AFR remains very low at 0.44, with marked intra-regional inequity (Seychelles, 4.297; South Sudan 0.003). The stock of dentists just exceeds that of dental assistants/therapists (1:0.91). Workforce density of dentists and the OHWF overall was strongly associated with the HDI and mean years of schooling. The dominant perceived challenge was identified as ‘mal-distribution of the workforce (urban/rural)’ and ‘oral health’ being ‘considered low priority’. Action to ‘strengthen oral health policy’ and provide ‘incentives to work in underserved areas’ were considered important solutions in the region. Whilst utilising workforce skill mix contributes to overall capacity, there is a stark deficit of human resources for oral health in the AFR. There is an urgent need to strengthen policy, health, and education systems to expand the OHWF using innovative workforce models to meet the needs of this region and achieve Universal Health Coverage (UHC).

## 1. Introduction

Epidemiological research highlights the burden of untreated oral disease in the World Health Organization (WHO) African Region (AFR) [1,2,3], with about half its population suffering from oral diseases, most notably dental caries, periodontal disease, and tooth loss [4]. Whilst most countries in the AFR have traditionally reported a lower caries risk than middle- and higher-income countries, in line with a traditional diet [1,5], nutritional transition to a Western diet has been linked to an increased prevalence of caries [6], even in the presence of hunger and malnutrition. The consequences of oral and dental disease are particularly acute in the AFR. Preventable fatalities associated with acute dental infections are of specific concern [7].

Additionally, there is increasing prevalence of dental trauma in both primary and secondary dentitions [1], variable levels of oral cancer (lip and oral cavity; age-standardized incidence rate of oral cavity cancer of 1.8 per 100,000 population) [5], and HIV with oral manifestations [5]. Cleft lip and/or palate are among the most frequent congenital disorders [1,2,5]. Furthermore, conditions such as Burkitt’s lymphoma [7] and noma [5] present in certain lower-income areas. 

Most oral and dental conditions are preventable, and/or treatable, particularly early in the disease process and should be identified and managed in primary care settings. Moreover, they share common risk factors with other noncommunicable diseases (NCDs), including diet (sugar), tobacco, alcohol, and poor hygiene [8,9]. Strategically, a common risk factor approach is vitally important, together with ensuring optimal fluoride delivery [10], whilst addressing underlying psychosocial and economic determinants [11]. 

National disease management, however, remains limited and vertical, rather than using integrated cost-effective strategies [1,2,3,5]. Furthermore, oral health has a low priority, as demonstrated by the absence of oral health policies, resources, and technical capacity [8,11]. Thus, an AFR Regional Oral Health Strategy 2016–2025 [8] was endorsed, guiding member states to integrate oral health into NCD-prevention and control in the context of universal health coverage (UHC) [11]. Globally in an historical turning point, the 74 [12], and 75th [13] World Health Assemblies have recognised the immense burden created by oral diseases, especially for low-income settings, including ‘to develop innovative workforce models to respond to population oral health needs’ as a key strategic pillar.

The AFR consist of 47 countries: Algeria, Angola, Benin, Botswana, Burkina Faso, Burundi, Cabo Verde, Cameroon, Central African Republic, Chad, Comoros, Congo, Cote d’Ivoire, Democratic Republic of Congo, Equatorial Guinea, Eritrea, Eswatini, Ethiopia, Gabon, Gambia, Ghana, Guinea, Guinea-Bissau, Kenya, Lesotho, Liberia, Madagascar, Malawi, Mali, Mauritania, Mauritius, Mozambique, Namibia, Niger, Nigeria, Rwanda, Sao Tome and Principe, Senegal, Seychelles, Sierra Leone, South Africa, South Sudan, Togo, Uganda, United Republic of Tanzania, Zambia, and Zimbabwe. The region largely comprises low- and middle-income states [14] and is recognised as the most challenged region globally, with limited health workforce capacity [15]. Therefore, the regional strategy [8], guides member states to take the following actions:

Promote capacity building in oral health promotion and integrated disease prevention and management for oral health professionals and other health and community workers matching the oral health needs of the population;

Develop workforce models for integration of basic oral healthcare within primary healthcare (PHC) [8].

In light of population needs and in support of current strategies [8,9,11,12,13], it is of the utmost importance to profile the oral health workforce (OHWF) in the AFR to inform action. Thus, the aim of this paper is to review the OHWF in the AFR, taking account of wider determinants, exploring contemporary challenges and possible solutions. 

The objectives are:

1. To describe the nature of the OHWF, density, trends, distribution, and education (relevant to training oral healthcare providers).
2. To examine the association between OHWF density, country level development, and urban/rural population distribution.
3. To explore OHWF challenges and possible solutions.

## 2. Materials and Methods

OHWF data were obtained from two sources. First, cross-sectional data from a global OHWF service evaluation survey of member states conducted by the WHO in collaboration with King’s College London, United Kingdom (UK), between January and December 2019. The scope of the survey was informed by the literature and the WHO workforce categories: dentists (2261), dental assistants/therapists (3251), and dental prosthetic technicians (3214) (Appendix A), based on the International Standard Classification of Occupations (ISCO-08) [16]. The survey was co-designed, approved, and conducted through WHO offices and comprised open and closed questions across four domains: capacity, capability and governance, education and training, and workforce challenges/solutions (Appendix B). Second, workforce volume and density per 10,000 population, over time, were extracted from the National Health Workforce Accounts (NHWA) [17] for dentists, dental assistants/therapists, and dental prosthetics/technicians. Density per 10,000 population involves dividing the number of reported professionals by the country’s population [18] (Appendix C).

This integrated study database was created through triangulation of data obtained from the above two sources for the 47 countries of the AFR. The latest reports from member states ranged from 2002 to 2019. Data management was performed using Microsoft Excel 365. Where workforce numbers were similar, the NHWA data were used. Where marked differences existed for the same year, the WHO authors liaised with the relevant member states to identify the most reliable data to include for analysis. 

### 2.1. Dataset 

Data on dentists were available for all AFR countries (100%; n = 47). Data coverage for the rest of the OHWF was lower, with 41 countries (87.2%) for dental assistants/therapists and 42 (89.4%) for dental prosthetics/technicians. Countries with missing data on their OHWF were excluded from analysis. 

Whilst most African countries (n = 39, 83%) updated their dentist numbers during 2018 and 2019, data on dental assistants/therapists and dental prosthetics/technicians, tended to be older and updated less frequently (64% of dental assistant/therapists; 66% dental prosthetics/technicians).

### 2.2. Data Analysis

First, OHWF density, trends, distribution, and education were examined. Descriptive analysis was performed to examine data coverage, workforce densities per 10,000 population, and the human development index (HDI). HDI provides information about a country’s education, health, longevity, and income [19]. 

Second, country-level association between OHWF density, development, and urban/rural population distribution was assessed to review the relationship with the national status and population distribution within a country. 

Third, unadjusted associations between density of dentists and density of the combined OHWF with ‘HDI’, ‘mean years of schooling’, ‘number of dental schools’, and ‘percentage of urban population’ were assessed to predict density of dentists and combined workforce through linear regression models for the countries included in the AFR. Previous studies have shown evidence that similar variables are predictors for NCD incidence [20,21,22] and for health workforce [23]. As densities of dentists and combined OHWF were continuous variables, linear regression was chosen as the most appropriate method of analysis. Its standard equation is demonstrated below:

Y = a + bX, where X is the explanatory variable and Y is the dependent variable was used.

In this model, population data and urbanization rates were obtained from the United Nations [18] and the World Bank [24]. Levels of urbanization are related to the presence of developed infrastructure and opportunities for employment after graduation [5]. The predefined distribution categories of HDI were used to describe each country: low (HDI < 0.5), medium (0.5 ≤ HDI < 0.7), high (0.7 ≤ HDI < 0.8), and very high (HDI ≥ 0.8) [19]. The Statistical Package for Social Sciences version 27 (SPSSv27) was used to perform this analysis. 

Fourth, and finally, qualitative data obtained from the global survey on OHWF challenges and possible solutions were examined by the research team descriptively and thematically [25] for member state perspectives.

## 3. Results

There were 36,222 dentists, 32,783 dental assistants/therapists, and 11,986 dental prosthetics/technicians reported for the AFR in 2019. Average density per 10,000 population was 0.44 for dentists, 0.39 for dental assistants/therapists, and 0.10 for dental prosthetics/technicians. The ratio of dentists per dental assistants/therapist was almost equivalent at 1: 0.91, whilst dentists per dental prosthetics/technician was 1:0.33. Countries with the lowest densities were South Sudan for dentists (0.003), Ethiopia for dental assistants/therapists (0.007), and Burkina Faso and the Democratic Republic of Congo (DRC), for dental prosthetic technicians (both 0.001). The Seychelles at 4.297 dentists per 10,000, 3.776 dental assistants/therapists, and 1.432 dental prosthetic technicians had the highest densities for all groups. 

Whilst 11 countries reported having no dental school, 22 countries reported the presence of 61 schools, with evidence of separate training colleges for other OHWF members. 

**Figure 1:**
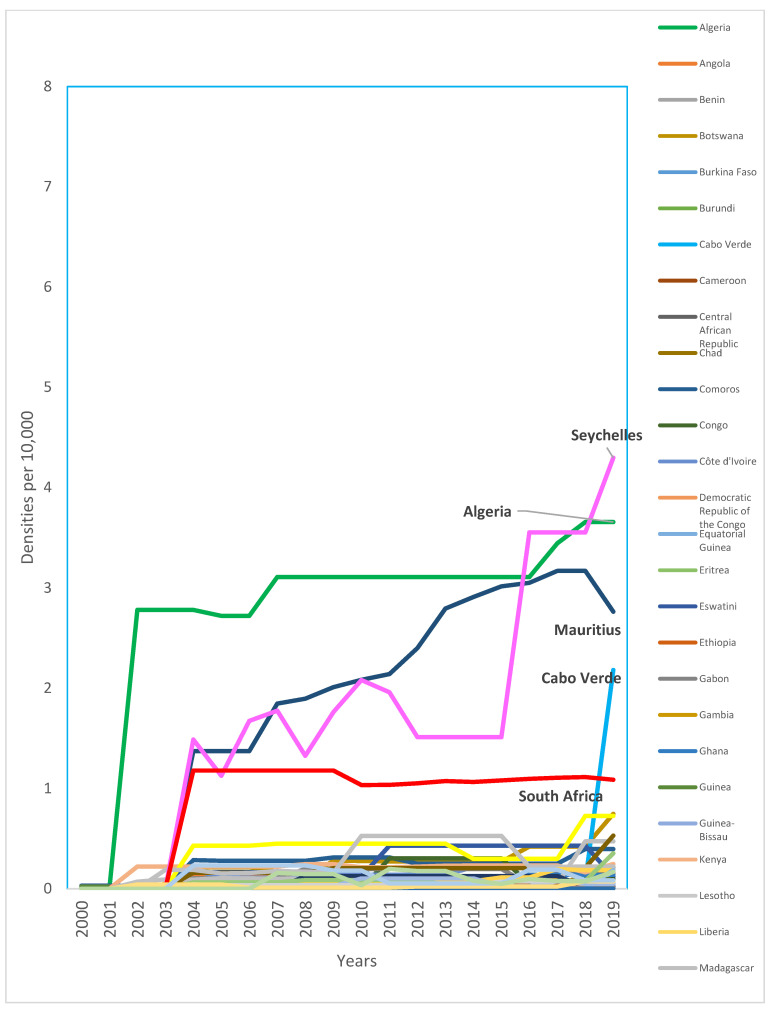
Trends in density of dentists per 10,000 population for the AFR, 2000-2019 (Appendix D). Source: The workforce data are based on the latest available data in the NHWA data platform as of 31 March 2021, apart from the data for 2019, which used a combination of the latest available data from the NHWA data platform and King’s College London survey. Countries with the highest densities were named in the plot and highlighted in bold.

Despite an overall growth of 63.6% in the density of dentists from 2010 (0.28 per 10,000) to 2019 (0.44, per 10,000), the density is consistently very low. Over the last decade, only four countries consistently exceeded one dentist per 10,000 population (Algeria, Mauritius, Seychelles, and South Africa) (Figure 1) (Appendix D). Available data suggest a higher rate of growth of dental assistants/therapists (238%) and dental prosthetic technicians (250%), with Nigeria having the highest growth for both groups. 

**Figure 2:**
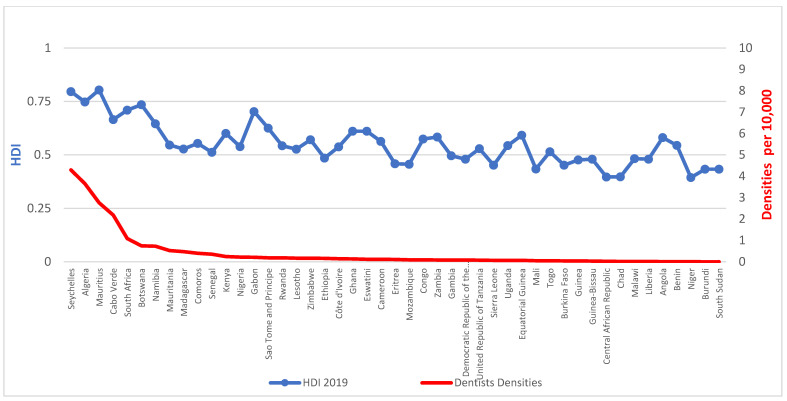
Densities of dentists per 10,000 population and Human Development Index (HDI) for the AFR, 2019. Source: The workforce data are based on a combination of the latest available data from the NHWA data platform and King’s College London survey. HDI was collected from the latest Human Development Report from the United Nations Development Programme (UNDP).

**Figure 3:**
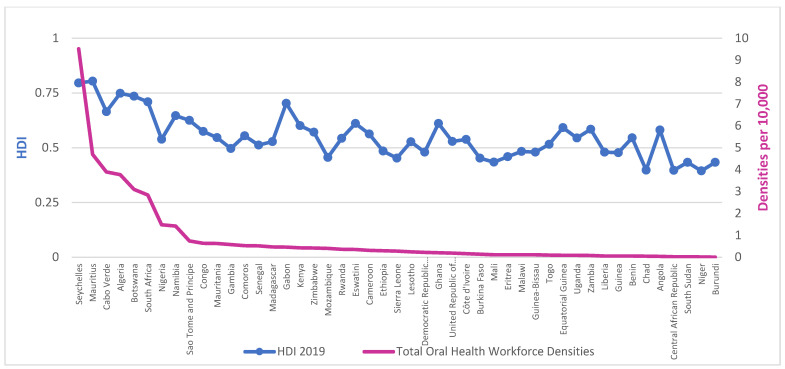
Densities of combined OHWF per 10,000 population and Human Development Index (HDI) for the AFR, 2019. Source: The workforce data are based on a combination of the latest available data from the NHWA data platform and King’s College London survey. HDI was collected from the latest Human Development Report from the United Nations Development Programme (UNDP).

Workforce densities in relation to HDI, presented in Figure 2 and Figure 3, demonstrate a clear social gradient. The average density of dentists per 10,000 population in countries with low HDI is 0.051 against 0.652 for medium HDI or above, whilst for those of ‘high HDI or above’, it is 1.270. 

Linear regression analysis revealed that country-level HDI scores were associated with the density of dentists (Table 1) and the combined OHWF (Table 2). For each unit increase in HDI, there was an increase in density of dentists of 6.53 and in density of combined OHWF of 12.07. Similarly, average years of schooling also showed a significant, but weaker, association with workforce densities but not with the reported number of dental schools and level of urbanization in this unadjusted model. 

**Figure 4:**
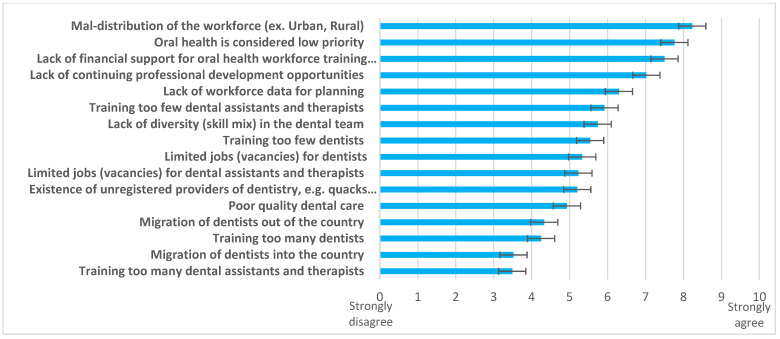
Challenges related to the OHWF highlighted by the AFR, 2019. Source: King’s College London survey, 2019.

**Figure 5:**
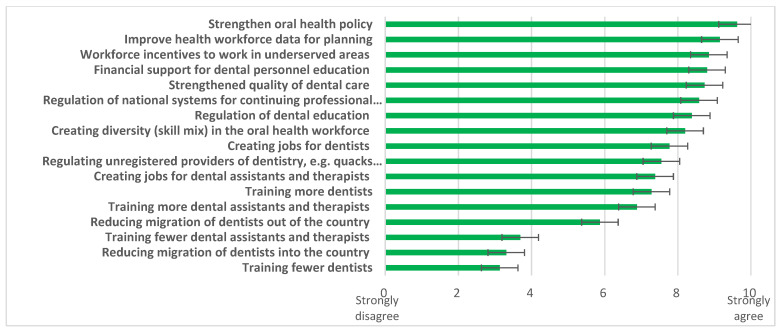
Possible solutions related to the OHWF highlighted by the AFR, 2019. Source: King’s College London & WHO Survey, 2019.

When challenges (Figure 4) and possible solutions (Figure 5) were explored, responding countries (n = 40) identified “*maldistribution of the workforce (urban/ rural)*”, “*oral health considered low priority*”, and “*lack of financial support for oral health workforce training institutions*” as the three biggest challenges. Multiple solutions were supported, led by the need to “*strengthen oral health policy*”, “*improve health workforce data for planning*”, and provide “*workforce incentives to work in underserved areas*”. 

Other reported challenges displayed common themes. First there were concerns posed over the “*lack of leadership at national level for oral health*”. Second, there were issues relating to education and training programmes and numbers, where the “*lack of dental auxiliaries such as therapists and assistants*” was identified and challenges regarding coverage given that there are “*landlocked areas not chosen by dentists*”. Third, lack of funding impacted the ability to provide patient care.

Comparable themes were displayed by 17 countries in highlighting additional solutions. Stronger governance was advocated to support action towards an appropriate OHWF, including *“defining clearly, the roles and responsibilities of the dental auxiliaries”* was advocated. Workforce redistribution required action, recognising that *“dentists and other oral health professionals congregate in cities leaving remote areas underserved”* were highlighted. To deliver this change, there was a great need identified by some countries to establish, extend, or re-establish dental education and training, including *“creating a dental school in the country, training existing staff, continuing training, implementing specialties”*, *“re-opening training schools”*, and enabling *“inclusion of essential oral health care services as part of UHC initiatives.”* Additional factors included enriching the system through *“exchanges with other countries”*, *“developing postgraduate training”*, and undertaking *“planning and operational research”.*

## 4. Discussion

To the knowledge of the authors, this is the first paper to comprehensively examine the contemporary OHWF in the AFR. The findings highlight the critically low levels to serve a region with a rapidly growing population, where workforce densities are strongly associated with the level of development (HDI) in particular. There is, however, evidence of embracing a workforce skill mix in similar proportions to dentists. Reported challenges include workforce maldistribution, exacerbated by a lack of financial support for training and workforce data for planning. Strengthening oral health policy and gaining better workforce data were perceived as important, together with having incentives to work in underserved areas to meet population oral health needs.

The average density of the OHWF for the AFR is 0.780 per 10,000 population (0.44 of which relates to dentists). For a continent where significant population growth is predicted [18], urgent action is needed to solve an imminent oral health crisis and address increasing levels of oral disease [5,8,26]. Europe, in contrast, with less than two-thirds of the population has a 12-times larger OHWF [17] and little predicted growth [18].

HDI [19], was a good predictor of dentist density; and, to a lesser extent, average years of schooling. Whilst there is also evidence that HDI is a predictor for NCD incidence [20,21,22], our findings support the view that it is a health workforce predictor [23]. Despite rapid expansion of higher education in recent years, shortages of high-skilled workers within certain fields, such as medicine, engineering, and dentistry, are a harsh reality [5,15,27]. It has been suggested that further expansion of higher education may need to be more selective to ensure a better match with labour market needs [27], but this is not the only solution as discussed below.

The global influence of traditional American and European models of oral and dental care, which focus on training dentists, has been significant. Despite the philosophical barrier these models may inflict on the development of the OHWF in low- and middle-income countries, several African states have started initiatives to shift the paradigm with successful examples of expansion and use of mid-level providers [7,28,29,30,31]. In line with the UHC targets [32], there is an urgent commitment to focus on preventative actions and social determinants of health to address oral health needs, as oral diseases are preventable in nature. For this, strong leadership is paramount. Higher education funding continues to be a challenge in sub-Saharan Africa despite recent growth [27]. Where evidence exists, it suggests shortages of high-skilled workers within certain fields of study, such as healthcare, may coexist with the rapid expansion of the sector, reflecting mismatches with the labour market [5].

Achieving UHC requires an appropriately educated and trained workforce with a relevant skill mix across community and primary care. A recent technical paper suggests that a seven-fold increase in dentists and dental assistants/therapists is required in the AFR to achieve 70% UHC [32]. Given the enormity of the task, the cost of training dentists and the need to develop a supportive workforce that will work in rural settings, where an estimated 59% of the population reside [24], capacity building should involve shifting from a dentist-centred model towards a diverse, yet integrated, approach [5,8,26]. Interestingly whilst certain Western countries have embraced a wider OHWF skill mix [33,34,35], the AFR is ahead with almost a 1:1 ratio. The role of mid-level providers in delivering healthcare generally has been advocated for the AFR [5], embraced by countries such as Rwanda [29,30], and being explored in Sierra Leone [7,31], Tanzania [36], and Malawi [37] in line with workforce research and guidance [11,38]. Integrating oral health within AFR primary healthcare (PHC) [5,8], whereby community health workers deliver most essential basic oral healthcare [5], with the latter acting as entry points to the health system, will clearly be vitally important for the future. The new WHO global competency and outcomes framework [39] for pre-service health professional training will thus be an important tool. It supports the building of a cadre of community health workers that can promote health, prevent disease, and be the gatekeepers to health services. The Regional Office for Africa has been developing competency-based e-training on oral health for community health workers [40]. In line with this approach, a recent oral health cascade training programme in Malawi [28], delivered by dental therapists trained with international support, has led to oral health promotion by community health workers in rural settings. As mentioned in the FDI World Dental Federation Vision 2030 [41], this is a prime example of co-development with upstream inter-governmental agreement, and a helpful model for supporting or facilitating future change to a more sustainable workforce in Africa [28,41]. Establishing indispensable health worker competencies to guide competency-based education [39,42] supports UHC.

Thus, innovative approaches to education and training that focus on community needs and evidence-informed care pathways, whereby care can be flexibly provided in an integrated manner [43], are essential to tackle the current crisis in the healthcare workforce. Ellard et al., suggest that whilst enhancing knowledge, practical skills, and clinical leadership of mid-level providers in the role of ‘associate clinicians’ may have a positive impact on health outcomes, ‘impact may be confounded by the significant challenges in delivering a service in terms of resources’ [44]; thus, ensuring appropriate resources as highlighted by the response of member states is important for workforce retention.

One of the main strengths of this study was the good response of member states to the survey which, together with the NHWA, provided good data for the AFR. This may possibly be explained by the importance of addressing this critical issue for responding states. However, despite achievements in terms of highlighting OHWF capacity, the focus remains on dentists, and there may be underreporting of mid-level personnel. Greater emphasis should be placed on the wider members of the OHWF team, and this is generally only possible when registration to practise is required, as most will not be employed in the state sector.

This work had the following limitations which should be recognised. First, for some countries, the latest available data on dental assistants and therapists and dental prosthetic technicians are over five years old. Second, not every country provided information on auxiliary members, and the absence of data does not mean that type of workforce is not available or provide insight to their scope of practice. Third, only external validation of data [45] was employed during the development of this research. Fourth, there is no evidence on the number of unregistered OHWF members and no data on the distribution of the workforce across rural and urban settings. Fifth, there is no consistent data on the number and structure of training institutions such as dental schools and training colleges. Sixth, there is no evidence on the working hours for the OHWF (part-time or full-time workers). Seventh, there is no evidence on coverage within a country given that in many settings, the OHWF is concentrated in urban areas, particularly the capital city [7]. Eighth, and finally, given the time lag in reporting data to the NHWA, obtaining contemporary data remains a challenge, particularly in this para-COVID environment.

Nonetheless, the research team created the most comprehensive dataset on the OHWF in Africa; data which need to be kept up-to-date and extended. The immense challenges of the COVID-19 pandemic have deepened oral health inequalities amongst the poorest and most vulnerable populations [46]. Healthcare institutions around the globe are exploring more efficient ways to deliver oral healthcare to people in need [47]. Countries should further strengthen capacity for OHWF data collection and reporting in support of health and planning [15,17,48]. Grouping all mid-level oral health professionals with distinct scope of practice under the umbrella of ‘dental assistants and therapists’ is not particularly helpful, especially for the African context, given variations in the scope of practice amongst mid-level providers [38] of oral health, such as dental assistants, dental nurses, dental hygienists, and dental therapists [30,35,37,49,50]. The OHWF in the AFR is very scarce [5,8]; therefore, being able to plan their distribution and training with greater precision and tailoring their development according to the location needs would be an important step to minimise the burden of oral health diseases of the region. More granularity for each workforce category and annual updates through NHWFA will be important to strengthen data quality and assist strategic planning.

In summary, there is an urgent need to develop primary care services with an appropriate workforce to address the pain, suffering, and even death from acute dental conditions [1,5,7]. Given the size of the challenge, there is great scope for innovative models of care [51,52] within a public health approach involving upstream action. Workforce capacity building should be contextually appropriate to deliver UHC in a sustainable manner rather than merely following traditional dentist-focused approaches. Greater collaboration has been strongly recommended to support capacity building [15,39,53], ideally working in partnership (involving co-production) [11,28,31,52] with successful examples emerging [29,36,37]. African countries may explore working together creatively to rethink the shape of health systems specific to their current needs and share learning. The recent publication of country profiles as part of the Global Oral Health Status Report provides important information to support all nations in preparing for effective action [54].

## 5. Conclusions

There is a stark oral health workforce deficit in the AFR; thus, there is an urgent need for developmental action supported by capacity building to address oral health needs, with great potential to develop innovative models of care which further utilise the workforce skill mix and community health personnel. This should be supported by oral health policy and health systems strengthening in support of achieving UHC.

## Figures and Tables

**Table 1:** Unadjusted linear regression analysis of dentists per 10,000 population in the AFR, 2019.

	Coefficient	Sig.	95% Conf. Interval
Human Development Index [HDI]	6.534	<0.001 *	4.670, 8.399
Mean Years of Schooling	0.231	<0.001 *	0.124, 0.337
Number of dental schools	0.050	0.522	−0.108, 0.209
% Urban population	0.009	0.217	−0.005, 0.022

* Statistically significant. Source: The workforce data are based on the latest available data in the NHWA data platform as of 31 March 2021, apart from the data for 2019, which uses a combination of the latest available data from the NHWA data platform and King’s College London survey. Linear regression analysis was performed in the Statistical Package for the Social Sciences (SPSS) 27 version.

**Table 2:** Unadjusted linear regression analysis of combined OHWF per 10,000 population in the AFR, 2019.

	Coefficient	Sig.	95% Conf. Interval
Human Development Index [HDI]	12.072	<0.001 *	8.588, 15.577
Mean Years of Schooling	0.460	<0.001 *	0.268, 0.652
Number of dental schools	0.022	0.880	−0.274, 0.318
% Urban population	0.016	0.210	−0.010, 0.042

* Statistically significant. Source: The workforce data are based on the latest available data in the NHWA data platform as of 31 March 2021, apart from the data for 2019, which uses a combination of the latest available data from the NHWA data platform and King’s College London survey. Linear regression analysis was performed in the Statistical Package for the Social Sciences (SPSS) 27 version.

**Figure A1:**
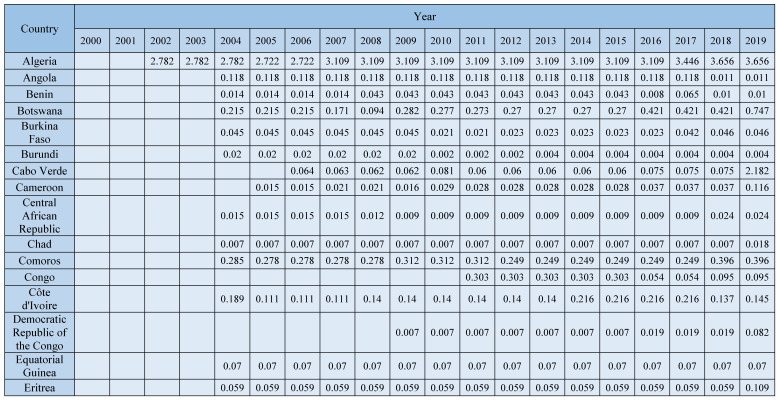

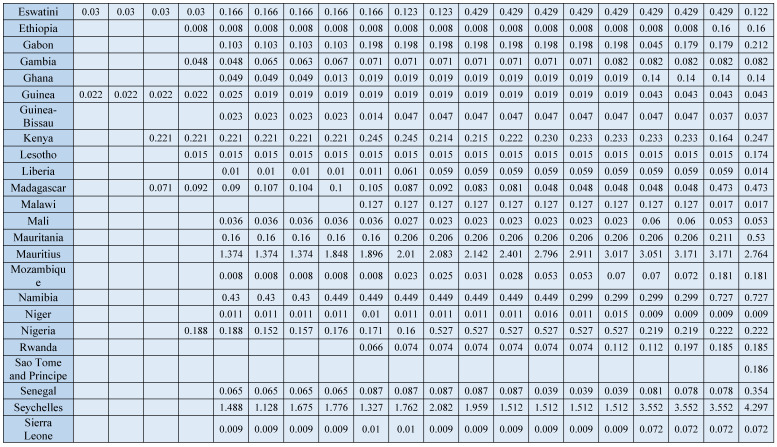

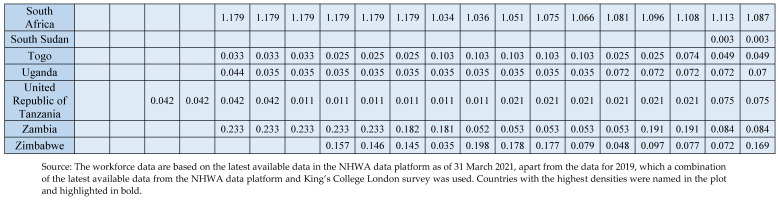
Trends in density of dentists per 10,000 population for the AFR, 2000–2019.

## Data Availability

Data available in a publicly accessible repository that does not issue DOIs. Publicly available datasets were analyzed in this study https://apps.who.int/nhwaportal/Home/Index (accessed on 10 March 2021). Data is contained within the article or in the Appendices A–D.

## Glossary

Accreditation	Accreditation is a process by which an officially approved body, on the basis of assessment of learning outcomes and /or competences according to different purposes and methods, awards qualifications (certificates, diplomas or titles), or grants equivalences, credit units or exemptions, or issues documents such as portfolios of competences. In some cases, the term accreditation applies to the evaluation of the quality of an institution or a programme as a whole.
Active health worker	Active health workers are those who provide services for patients (practising health professionals). In case of data not available for practising health workers, data closest to practising (professionally active health workers, health workers with active license) can be used.
Community Health Workers	Community health workers provide health education, referral and follow-up, case management, basic preventive healthcare and home visiting services to specific communities. They provide support and assistance to individuals and families in navigating the health and social services system.
Continuing professional development	Training that is beyond clinical update and includes wide-ranging competencies like research and scientific writing; multidisciplinary context of patient care; professionalism and ethical practice; communication, leadership, management and behavioural skills; team building; information technology; auditing; and appropriate attitudinal change to ensure improved patient service and research outcomes and attainment of the highest degree of satisfaction by stakeholders.
Dentist	Dentists diagnose, treat, and prevent diseases, injuries, and abnormalities of the teeth, mouth, jaws, and associated tissues by applying the principles and procedures of modern dentistry. They use a broad range of specialized diagnostic, surgical, and other techniques to promote and restore oral health.
Dental assistants and therapists	Dental assistants and therapists provide basic dental care services for the prevention and treatment of diseases and disorders of the teeth and mouth, according to care plans and procedures established by a dentist or other oral health professional. Dental hygienists and dental nurses are included in this category.
Dental aids	Key dental personnel who are involved in infection control, organize dental surgery, prepare, mix, and handle dental materials, provide chairside support to the operator during treatment. Dental surgery assistants and dental nurses (non-clinical) are included in this category.
Dental Prosthetic/Technician	Dental prosthetic technicians design, fit, service, and repair dental devices and appliances following prescriptions or instructions established by a health professional. They may service a wide range of support instruments to correct dental problems, such as dentures, dental crowns, and bridges. This will include clinical and non-clinical technicians.
Foreign-trained dentists	Health workers who have obtained their first medical qualification (degree) in another country and are entitled to practise in the receiving country.
Inter-professional education system	Inter-professional education occurs when two or more health professionals learn about, from, and with each other to enable effective collaboration and improve health outcomes. Professional is an all-encompassing term that includes individuals with the knowledge and/or skills to contribute to the physical, mental, and social well-being of a community.
License, certificate	The license or certification is the permission to practise in the appropriate field of health and is issued by a legitimate regulatory body within the profession.
Skill mix	A relatively broad term that can refer to the mix of staff in the workforce or the demarcation of roles and activities among different categories of staff.

## Appendix A

Definition of the oral health workforce categories

Dentist (2261)	Dentists diagnose, treat, and prevent diseases, injuries, and abnormalities of the teeth, mouth, jaws, and associated tissues by applying the principles and procedures of modern dentistry. They use a broad range of specialised diagnostic, surgical, and other techniques to promote and restore oral health.
Dental Assistants and Therapists (3251)	Dental assistants and therapists provide basic dental care services for the prevention and treatment of diseases and disorders of the teeth and mouth, according to care plans and procedures established by a dentist or other oral health professional. Dental hygienists and dental nurses are included in this category.
Dental Prosthetic/Technician (3214)	Dental prosthetic technicians design, fit, service, and repair dental devices and appliances following prescriptions or instructions established by a health professional. They may service a wide range of support instruments to correct dental problems, such as dentures, dental crowns, and bridges. This will include clinical and non-clinical technicians.
Source: [16].	

Glossary of common terms related to oral health workforce

Mid-level oral health workforce providers	Mid-level oral health workers are those who have received shorter training than dentists (between 2–4 years) but will perform some of the same tasks as dentists. Therefore, a mid-level oral health worker is not a clinical dentist but is able to provide clinical care (may diagnose, manage, and treat disease and impairments) or engage in preventive care and health promotion. The examples are dental assistants, dental nurses, dental prosthetists, dental therapists, and dental hygienists.
Sources: [11,38,55].	

Task shifting	Task shifting is defined as delegating tasks to existing or new health workers with either less training or narrowly tailored training. It involves the rational redistribution of tasks among health workforce teams. Specific tasks are moved, where appropriate, from highly qualified cadres to cadres with shorter training and fewer qualifications in order to make more efficient use of the available human resources for health.
Source: [56].	

World Health Organization, 2018. Mid-level health workers: a review of the evidence. https://apps.who.int/iris/handle/10665/259878 (accessed on 8 December 2022).

Skill mix	Skill mix is a broad term that has been defined as the proportion of staff qualifications, levels of competence, abilities, knowledge, and experience that are necessary to achieve an agreed upon standard of care for a given level of demand. It represents the mix of different types of staff in a team/healthcare setting.
Sources: [57,58].	

## Appendix B

### Appendix B.1. Global Survey

The WHO Global Oral Health Workforce Survey

Start of Block: Introduction

The WHO Global Oral Health Workforce Survey Purpose: The purpose of this survey is to gauge your country’s workforce information responding to oral health. It will provide the global situation of oral health workforce to describe current workforce situation and provide clear picture how to leverage existing system (health workforce) to maximize population benefit, health and well-being at country level in the current global context, namely, Universal Health Coverage (UHC) and the 13th General Programme of Work towards the Sustainable Development Goals (SDGs).

The information collected through this survey will be presented as part of the “Global Oral Health Report” which is planned to be launched in May 2020.

The results of this survey will also be contributed to the WHO National Health Workforce Accounts.

Use of standardized questions allows comparisons of country capacities and responses.

We have divided this survey into four modules:

General information

Oral health workforce: capacity, capability and governance

Education and training of the oral health workforce

Better use of the oral health workforce

Process:

A focal point or survey coordinate will need to be identified to coordinate and ensure survey completion. However, in order to provide a complete response, a group of respondents with expertise in the topics covered in the modules will be needed. Please use the table provided to indicate names and titles of all of those who have completed the survey and which sections they have completed. Please also add any additional information on other sources you may have consulted in developing your response.

Please note that while there is space to indicated “Don’t Know” for most questions, there should be very few of these. If someone is filling in numerous “Don’t Knows”, another person who is more aware of this information should be found to complete this section.

The responses are automatically saved and you can return back to the responses within 3 months if you access it through the same URL.

As far as possible, in order to validate responses, documentation will be requested for affirmative responses throughout the questionnaire. Please make every effort to provide electronic copies of the requested documentation by email (varenneb@who.int and makinoy@who.int). If you are unable to provide electronic copies through the email then please contact varenneb@who.int and makinoy@who.int for an alternative means to submit documentation. Please note that questionnaire survey terminology is aligned with the International Standard Classification of Occupations (ISCO-08) and the WHO National Health Workforce Accounts.

*[ISCO-08*: http://www.ilo.org/wcmsp5/groups/public/@dgreports/@dcomm/@publ/documents/publication/wcms_172572.pdf (accessed on 8 December 2022)

https://www.ilo.org/public/english/bureau/stat/isco08/index.htm (accessed on 8 December 2022)]

If you need help completing the survey at any point please contact: makinoy@who.int

Module 1: General information

1.1. Country

▼ Afghanistan (1) ... Zimbabwe (194)

1.2. Details of personnel completing the survey (please state Not Available (NA) where appropriate)

	**Responsible** **Officer Name (1)**	**Position (2)**	**Institution (3)**	**Email Address (4)**
All modules (1)				
Module 1 (6)				
Module 2 (7)				
Module 3 (8)				
Module 4 (9)				

End of Block: Section 1. You and Your Country

Start of Block: Section 2. Oral Health Workforce Overview

Module 2: Oral Health Workforce: capacity, capability and governance

Please fill in all of the blanks. If you do not know answer, or not available for your country please add Don’t Know (DK) or Not Available (NA).

2.1. Briefly, please list the types of oral health workforce (i.e., dentists, dental nurses, hygienists, therapists, technicians, etc.) working in your country?

2.2. Which organization, or organizations, hold dental workforce data in your country?

2.3. Which oral health workforce are working in your country, how many of them are there currently, and are they regulated? Taking each one in turn, please provide the following information on numbers. (Please complete all relevant boxes and answer as best you can)

2.3.1. Which oral health workforce are working in your country and how many of them are there currently?

2.3.1.1. Dentists

	**Size of Workforce**		
	**Number (1)**	**Is this an Estimate** **(State YES or NO) (2)**	**Year of Data (3)**
Dentists (1)			

Dentists diagnose, treat and prevent diseases, injuries and abnormalities of the teeth, mouth, jaws and associated tissues by applying the principles and procedures of modern dentistry. They use a broad range of specialized diagnostic, surgical and other techniques to promote and restore oral health.

2.3.1.2. Dental assistants and therapists

	**Size of Workforce**		
	**Number (1)**	**Is This an Estimate** **(State YES or NO) (2)**	**Year of Data (3)**
2a. Dental therapists (2)			
2b. Dental hygienists (3)			
2c. Dental assistants (11)			
2d. Dental nurses (4)			

Q46 Dental assistants and therapists provide basic dental care services for the prevention and treatment of diseases and disorders of the teeth and mouth, according to care plans and procedures established by a dentist or other oral health professional. Under this dental assistants and therapists, dental hygienists and dental nurses (clinical) will be covered.

2.3.1.3. Dental prosthetic technicians

	**Size of Workforce**		
	**Number (1)**	**Is This an Estimate** **(State YES or NO) (2)**	**Year of Data (3)**
Dental prosthetic technicians (6)			

Dental prosthetic technicians design, fit, service and repair dental devices and appliances following prescriptions or instructions established by a health professional. They may service a wide range of support instruments to correct dental problems, such as dentures, and dental crowns and bridges. This will include clinical and non-clinical technicians.

2.3.1.4. Dental aides

	**Size of Workforce**		
	**Number (1)**	**Is This an Estimate ** **(State YES or NO) (2)**	**Year of Data (3)**
Dental aides (12)			

Dental aides are key dental personnel who are involved in infection control, organization of the dental surgery, prepare, mix and handle dental materials, provide chair side support to the operator during treatment. Under this dental aides, dental surgery assistants and dental nurses (non-clinical) will be covered.

2.3.1.5. Other (if your country has other oral health workforce, please specify here. If not, please add NA (not available))

	**Size of Workforce**		
	**Number (1)**	**Is This an Estimate** **(State YES or NO) (2)**	**Year of Data (3)**
Other (11)			

2.3.2. If permission (license, certificate, registration) is required to practice, please select appropriate responsible bodies from the drop down list based on your country context.

	**License/Certificate**	**Registration**
1. Dentists (1)	▼ Government ex. MOH (1 ... Don’t Know (5)	▼ Government ex. MOH (1 ... Don’t Know (5)
2a. Dental therapists (2)	▼ Government ex. MOH (1 ... Don’t Know (5)	▼ Government ex. MOH (1 ... Don’t Know (5)
2b. Dental hygienists (3)	▼ Government ex. MOH (1 ... Don’t Know (5)	▼ Government ex. MOH (1 ... Don’t Know (5)
2c. Dental assistants (8)	▼ Government ex. MOH (1 ... Don’t Know (5)	▼ Government ex. MOH (1 ... Don’t Know (5)
2d. Dental nurses (4)	▼ Government ex. MOH (1 ... Don’t Know (5)	▼ Government ex. MOH (1 ... Don’t Know (5)
3. Dental prosthetic technicians (5)	▼ Government ex. MOH (1 ... Don’t Know (5)	▼ Government ex. MOH (1 ... Don’t Know (5)
4. Dental aides (10)	▼ Government ex. MOH (1 ... Don’t Know (5)	▼ Government ex. MOH (1 ... Don’t Know (5)
5. Other (7)	▼ Government ex. MOH (1 ... Don’t Know (5)	▼ Government ex. MOH (1 ... Don’t Know (5)

2.3.3. Are community health workers involved in oral health activities?

▼ Don’t Know (1) ... No (2)

2.3.4. If yes, what percentages of community health workers are involved in the oral health activities? (If no, then select don’t know)

▼ Don’t know (1) ... 99 (100)

2.4. What proportion of the oral health workforce as clinical practitioners are in respective sectors? Percentage of (active) health workers employed in facilities by type of ownership (public, private) [please ensure all boxes have been completed using the bottom control bar]

	**Public**		**Private**		**Public/Private Mix**		**Other (e.g., Non-profit Organizations such as NGOs, Church, etc.)**		**Inactive (e.g., Unemployed, Maternity Leave, Sabbatical Year, etc.)**	
	**% (1)**	**Number (2)**	**% (1)**	**Number (2)**	**% (1)**	**Number (2)**	**% (1)**	**Number (2)**	**% (1)**	**Number (2)**
1. Dentists (1)										
2a. Dental therapists (2)										
2b. Dental hygienists (3)										
2c. Dental assistants (4)										
2d. Dental nurses (5)										
3. Dental prosthetic technicians (6)										
4. Dental aides (10)										
5. Community health workers (7)										
6. Other (8)										

2.5. What is the gender distribution of ACTIVE dentists?

◦ Majority male (1)
◦ Majority female (2)
◦ 50/50 (3)
◦ Don’t know (4)

2.6. What is the gender distribution of ACTIVE dental assistants and therapists?

◦ Majority male (1)
◦ Majority female (2)
◦ 50/50 (3)
◦ Don’t know (4)

2.7. What is the age distribution of ACTIVE dentists? (answer in percentage)

a. Under 25 years (1)	▼ Don’t Know (1) ... 100 (101)
b. 25–34 years (2)	▼ Don’t Know (1) ... 100 (101)
c. 35–44 years (3)	▼ Don’t Know (1) ... 100 (101)
d. 45–54 years (4)	▼ Don’t Know (1) ... 100 (101)
e. 55–64 years (5)	▼ Don’t Know (1) ... 100 (101)
f. 65 years and above (6)	▼ Don’t Know (1) ... 100 (101)

2.8. What is the age distribution of ACTIVE dental assistants and therapists? (answer in percentage)

a. Under 25 years (1)	▼ Don’t Know (1) ... 100 (101)
b. 25–34 years (2)	▼ Don’t Know (1) ... 100 (101)
c. 35–44 years (3)	▼ Don’t Know (1) ... 100 (101)
d. 45–54 years (4)	▼ Don’t Know (1) ... 100 (101)
e. 55–64 years (5)	▼ Don’t Know (1) ... 100 (101)
f. 65 years and above (6)	▼ Don’t Know (1) ... 100 (101)

2.9. What is the proportion of ACTIVE foreign-trained dentists work in your country? (answer in percentage)

▼ Don’t know (1) ... 99 (100)

2.10. Does your country have dental specialties?

▼ Yes (4) ... No (5)

2.10.1. If yes, please list the types of dental specialists that practice in your country.

Module 3: Education and training of the oral health workforce

Please fill in all of the blanks. If you do not know answer, or not available for your country please add Don’t Know (DK) or Not Available (NA).

A. Dental Schools

	**Number (1)**	**Length of Course (Years) (3)**	**Comments (4)**
3.1 How many dental schools do you have in your country in total? (1)			
3.2 How many public dental schools in your country? (2)			
3.3 How many private dental schools in your country? (3)			

B. Dental Assistants and Therapists Schools (dental hygienists and clinical dental nurses are included in this group)

	**Number (1)**	**Length of Course (Years) (3)**	**Comments (4)**
3.4 How many schools for dental assistants and therapists are there in your country in total? (1)			
3.5 How many public schools for dental assistants and therapists in your country? (2)			
3.6 How many private schools for dental assistants and therapists in your country? (3)			

C. New graduates

	**Total Number (3)**	**Graduates Starting Practice within One Year (%) (4)**
3.7 How many dentist graduated last year from public dental schools? (1)			
3.8 How many dentist graduates last year from private dental schools? (2)			
3.9 How many dental assistants and therapists graduated last year from public schools? (3)			
3.10 How many dental assistants and therapists graduated last year from private schools? (4)			

Dentists specific questions

D. Dentists’ accreditation system and system for foreign-trained dentists

	**Yes (2)**	**No (3)**	**Don’t Know (4**)
3.11 Does your country have accreditation mechanisms for dental school education? (1)			
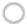
3.12 Does your country have formal mechanism for accreditation of diplomas/degrees obtained abroad? (2)			

E. Inter-professional education system

	**Yes (1)**	**No (2)**	**Don’t Know (3)**
3.13 Does your country have national and/or subnational standards for inter-professional education? (1)			
3.14 Does your country have a formal mechanism for the recognition of inter-professional education? (2)			

F. Existence of national systems for continuing professional development

	**Yes (1)**	**No (2)**
3.15 Do you have national systems for continuing professional development? (1)		
3.15.1 If yes, is it mandatory to participate to maintain licensing? If no, please proceed to the next module. (2)		

Module 4: Better use of the oral health workforce

4.1. Challenges related to oral health workforce in your country. To what extent do you agree with the following statements (strongly disagree [1] to strongly agree [10])?

	**Strongly Disagree**					**Strongly Agree**				
**...**	**1**	**2**	**3**	**4**	**5**	**6**	**7**	**8**	**9**	**10**
a. Training too many dentists ()										
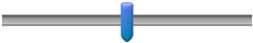
b. Training too few dentists ()										
c. Migration of dentists into the country ()										
d. Migration of dentists out of the country ()										
e. Training too many dental assistants and therapists ()										
f. Training too few dental assistants and therapists ()										
g. Limited jobs (vacancies) for dentists ()										
h. Limited jobs (vacancies) for dental assistants and therapists ()										
i. Lack of diversity (skill mix) in the dental team ()										
j. Mal-distribution of the workforce (ex. Urban, Rural) ()										
k. Poor quality dental care ()										
l. Lack of continuing professional development opportunities ()										
m. Existence of unregistered providers of dentistry, e.g., quacks and traditional healers ()										
n. Lack of financial support for oral health workforce training institutions ()										
o. Lack of workforce data for planning ()										
p. Oral health is considered low priority ()										
q. Other (please specify) ()										

4.2. Solutions related to oral health workforce in your country. To what extent do you agree with the following statements (strongly disagree [1] to strongly agree [10])?

	**Strongly Disagree**					**Strongly Agree**				
	**1**	**2**	**3**	**4**	**5**	**6**	**7**	**8**	**9**	**10**
a. Training more dentists ()										
b. Training fewer dentists ()										
c. Reducing migration of dentists out of the country ()										
d. Reducing migration of dentists into the country ()										
e. Training fewer dental assistants and therapists ()										
f. Training more dental assistants and therapists ()										
g. Creating jobs for dentists ()										
h. Creating jobs for dental assistants and therapists ()										
i. Workforce incentives to work in underserved areas ()										
j. Creating diversity (skill mix) in the oral health workforce ()										
k. Strengthened quality of dental care ()										
l. Regulating unregistered providers of dentistry, e.g., quacks and traditional healers ()										
m. Financial support for dental personnel education ()										
n. Improve health workforce data for planning ()										
o. Regulation of dental education ()										
p. Regulation of national systems for continuing professional development ()										
q. Strengthen oral health policy ()										
r. Other (please specify) ()										

4.3. How would you like to see dentistry changing in your country? E.g., skill mix, inter-professional working

THANK YOU FOR TAKING THE TIME TO ANSWER THIS QUESTIONNAIRE

As far as possible, in order to validate responses, documentation will be requested for affirmative responses throughout the questionnaire. Please make every effect to provide electronic copies of the requested documentation by email: varenneb@who.int and makinoy@who.int If you are unable to provide the electronic copies through the email, please contact varenneb@who.int and makinoy@who.int for an alternative means to submit documentation.

Going to the next page will submit the survey and you will not be able to return to previous questions after your submission.

## Appendix C

Data availability per country and professional category in the AFR.

**Countries**	**Dentists**	**DATs (Assistants and Therapists)**	**DPTs (Prosthetics Technicians)**
Algeria	√	√	√
Angola	√	√	
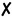
Benin	√	√	√
Botswana	√	√	√
Burkina Faso	√	√	√
Burundi	√		
Cabo Verde	√	√	√
Cameroon	√		
Central African Republic	√		√
Chad	√	√	√
Comoros	√	√	√
Congo	√	√	√
Cote d’Ivoire	√	√	√
Democratic Republic of the Congo	√		√
Equatorial Guinea	√	√	√
Eritrea	√	√	
Eswatini	√	√	√
Ethiopia	√	√	√
Gabon	√	√	√
Gambia	√	√	√
Ghana	√	√	√
Guinea	√		√
Guinea-Bissau	√	√	√
Kenya	√	√	√
Lesotho	√	√	√
Liberia	√	√	√
Madagascar	√	√	√
Malawi	√	√	
Mali	√	√	√
Mauritania	√	√	√
Mauritius	√	√	√
Mozambique	√	√	√
Namibia	√	√	√
Niger	√		√
Nigeria	√	√	√
Rwanda	√	√	√
Sao Tome and Principe	√	√	√
Senegal	√	√	√
Seychelles	√	√	√
Sierra Leone	√	√	√
South Africa	√	√	√
South Sudan	√	√	√
Togo	√	√	√
Uganda	√	√	√
United Republic of Tanzania	√	√	√
Zambia	√	√	√
Zimbabwe	√	√	√
Source: The workforce data are based on the latest available data in the NHWA data platform as of 31 March 2021, apart from the data for 2019, which a combination of the latest available data from the NHWA data platform and King’s College London survey was used. Available: ✓. Not available: ✗.			

## References

[1] Abid A., Maatouk F., Berrezouga L., Azodo C., Uti O., El-Shamy H., Oginni A. (2015). Prevalence and Severity of Oral Diseases in the Africa and Middle East Region. Adv. Dent. Res..

[2] Bernabe E., Marcenes W., Hernandez C.R., Bailey J., Abreu L.G., Alipour V., Amini S., Arabloo J., Arefi Z., GBD 2017 Oral Disorders Collaborators (2020). Global, Regional, and National Levels and Trends in Burden of Oral Conditions from 1990 to 2017: A Systematic Analysis for the Global Burden of Disease 2017 Study. J. Dent. Res..

[3] Elamin A., Garemo M., Mulder A. (2021). Determinants of dental caries in children in the Middle East and North Africa region: A systematic review based on literature published from 2000 to 2019. BMC Oral Health.

[4] Institute for Health Metrics and Evaluation (2022). GBD Results Tool. http://ghdx.healthdata.org/gbd-results-tool.

[5] World Health Organization Promoting Oral Health in Africa: Prevention and Control of Oral Diseases and Noma as Part of Essential Noncommunicable Disease Interventions 2016 [Updated 3 August 2021. 126]. https://apps.who.int/iris/handle/10665/205886.

[6] Folayan M.O., Oginni A.B., El Tantawi M., Alade M., Adeniyi A.A., Finlayson T.L. (2020). Association between nutritional status and early childhood caries risk profile in a suburban Nigeria community. Int. J. Paediatr. Dent..

[7] Ghotane S.G., Challacombe S.J., Gallagher J.E. (2019). Fortitude and resilience in service of the population: A case study of dental professionals striving for health in Sierra Leone. BDJ Open.

[8] World Health Organization. Regional Office for Africa (2016). Regional Oral Health Strategy 2016–2025: Addressing Oral Diseases as Part of Noncommunicable Diseases: Report of the Secretariat Brazzaville. https://www.afro.who.int/publications/regional-oral-health-strategy-2016-2025-addressing-oral-diseases-part-noncommunicable.

[9] World Health Organization (2021). Global Strategy on Tackling Oral Diseases: World Health Organization. https://www.who.int/teams/noncommunicable-diseases/governance/gaporalhealth.

[10] Godson J., Gallagher J. (2021). Delivering Better Oral Health 2021-What’s new and where next?. Community Dent. Health.

[11] World Health Organization (2022). Political Declaration of the Third High-Level Meeting of the General Assembly on the Prevention and Control of Noncommunicable Diseases: World Health Organization. https://apps.who.int/gb/ebwha/pdf_files/EB150/B150_7-en.pdf.

[12] World Health Organization (2021). 74th World Health Assembly, Resolution WHA74.5: World Health Organization, Geneva. https://apps.who.int/gb/ebwha/pdf_files/WHA74/A74_R5-en.pdf.

[13] World Health Organization (2022). 75th World Health Assembly, Documents A75/10. Geneva: World Health Organization. https://apps.who.int/gb/Journal/e/WHA75/JRN-A75-3_en.html.

[14] The World Bank (2021). Countries Income Status 2020–2021: The World Bank. https://datahelpdesk.worldbank.org/knowledgebase/articles/906519-world-bank-country-and-lending-groups.

[15] World Health Organization The African Regional Framework for the Implementation of the Global Strategy on Human Resources for Health: Workforce 2030: World Health Organization. 2020 [12p]. https://apps.who.int/iris/handle/10665/260238.

[16] International Standard Classification of Occupations (ISCO-08) (2012). Structure, Group Definitions and Correspondence Tables [Internet]. International Labor Office. https://www.ilo.org/wcmsp5/groups/public/---dgreports/---dcomm/---publ/documents/publication/wcms_172572.pdf.

[17] World Health Organization (2021). National Health Workforce Accounts Data Portal: World Health Organization. https://apps.who.int/nhwaportal/Home/Index.

[18] United Nations (2019). World Population Prospects 2019: Volume I: Comprehensive Tables: United Nations. https://population.un.org/wpp/publications/Files/WPP2019_Volume-I_Comprehensive-Tables.pdf.

[19] United Nations Development Programme (2021). Human Development Reports, Data Center- Human Development Index (HDI): United Nations Development Programme. http://hdr.undp.org/en/content/human-development-index-hdi.

[20] Roncalli A.G., Sheiham A., Tsakos G., de Araújo-Souza G.C., Watt R.G. (2016). Social factors associated with the decline in caries in Brazilian children between 1996 and 2010. Caries Res..

[21] Reda S.M., Krois J., Reda S.F., Thomson W.M., Schwendicke F. (2018). The impact of demographic, health-related and social factors on dental services utilization: Systematic review and meta-analysis. J. Dent..

[22] Herrera-Serna B.Y., Lara-Carrillo E., Toral-Rizo V.H., do Amaral R.C., Aguilera-Eguía R.A. (2019). Relationship between the human development index and its components with oral cancer in Latin America. J. Epidemiol. Glob. Health.

[23] Davies J.I., Vreede E., Onajin-Obembe B., Morriss W.W. (2018). What is the minimum number of specialist anaesthetists needed in low-income and middle-income countries?. BMJ Glob. Health.

[24] The World Bank (2021). Rural Population (% of Total Population): The World Bank. https://data.worldbank.org/indicator/SP.RUR.TOTL.ZS?locations=ZG.

[25] Ritchie J., Lewis J., Nicholls C.M., Ormston R. (2013). Qualitative Research Practice: A Guide for Social Science Students and Researchers.

[26] Watt R.G., Daly B., Allison P., Macpherson L.M., Venturelli R., Listl S., Weyant R.J., Mathur M.R., Guarnizo-Herreño C.C., Celeste R.K. (2019). Ending the neglect of global oral health: Time for radical action. Lancet.

[27] Majgaard K., Mingat A. (2012). Education in Sub-Saharan Africa: A Comparative Analysis.

[28] Bagg J. The MalDent Project: Oral Health for All [Internet]. Online 2022. [cited 2022]. https://themaldentproject.com/.

[29] Hackley D.M., Mumena C.H., Gatarayiha A., Cancedda C., Barrow J.R. (2018). A Case Study Optimizing Human Resources in Rwanda’s First Dental School: Three Innovative Management Tools. J. Dent. Educ..

[30] Morgan J.P., Isyagi M., Ntaganira J., Gatarayiha A., Pagni S.E., Roomian T.C., Finkelman M., Steffensen J.E., Barrow J.R., Mumena C.H. (2018). Building oral health research infrastructure: The first national oral health survey of Rwanda. Glob. Health Action.

[31] Ghotane S.G., Don-Davis P., Kamara D., Harper P.R., Challacombe S.J., Gallagher J.E. (2021). Needs-led human resource planning for Sierra Leone in support of oral health. Hum. Resour. Health.

[32] World Health Organization Regional Office for Africa (2021). Health Workforce Thresholds for Supporting Attainment of Universal Health Coverage in the African Region.

[33] Wanyonyi K., Radford D., Gallagher J. (2013). The relationship between access to and use of dental services following expansion of a primary care service to embrace dental team training. Public Health.

[34] Colonio Salazar F., Andiappan M., Radford D., Gallagher J. (2017). Attitudes of the first cohort of student groups trained together at the University of Portsmouth Dental Academy towards dental interprofessional education. Eur. J. Dent. Educ..

[35] Gallagher J.E., Lim Z., Harper P.R. (2013). Workforce skill mix: Modelling the potential for dental therapists in state-funded primary dental care. Int. Dent. J..

[36] England R., Paterson A., Jones A. (2021). Delivering urgent oral healthcare in Sub-Saharan Africa: Supporting sustainable local development. Prim. Dent. J..

[37] Paterson A., Chipanda M., Mukiwa W., Wood Y., Weil K., Wilson K., Passmore V., Milne V., Milne M., Jones A. Teaching Dental Therapists in Northern Malawi to Cascade Key Oral Health Messages to Remote and Rural Communities—A Pilot Project. Proceedings of the NHS Education for Scotland Dental Education.

[38] World Health Organization (2018). Mid-lLevel Health Workers: A Review of the Evidence: World Health Organization. https://apps.who.int/iris/handle/10665/259878.

[39] World Health Organization (2022). Global Competency and Outcomes Framework for Universal Health Coverage: World Health Organization. https://apps.who.int/iris/handle/10665/352711.

[40] World Health Organization Regional Office for Africa Filling the Gaps in Oral Health Services in Africa 2022 [Updated 11th June 2021. https://www.afro.who.int/news/filling-gaps-oral-health-services-africa.

[41] Glick M., Williams D.M. (2021). FDI vision 2030: Delivering optimal oral health for all. Int. Dent. J..

[42] World Health Organization (2022). Global Competency Framework for Universal Health Coverage.

[43] Fisher J., Selikowitz H.-S., Mathur M., Varenne B. (2018). Strengthening oral health for universal health coverage. Lancet.

[44] Ellard D.R., Shemdoe A., Mazuguni F., Mbaruku G., Davies D., Kihaile P., Pemba S., Bergström S., Nyamtema A., Mohamed H.M. (2016). Can training non-physician clinicians/associate clinicians (NPCs/ACs) in emergency obstetric, neonatal care and clinical leadership make a difference to practice and help towards reductions in maternal and neonatal mortality in rural Tanzania? The ETATMBA project. BMJ Open.

[45] Toresen Lokdam N., Riksheim Stavseth M., Bukten A. (2021). Exploring the external validity of survey data with triangulation: A case study from the Norwegian Offender Mental Health and Addiction (NorMA) Study. Res. Methods Med. Health Sci..

[46] Leon S., Giacaman R.A. (2020). COVID-19 and Inequities in Oral Health Care for Older People: An Opportunity for Emerging Paradigms. JDR Clin. Trans. Res..

[47] Benzian H., Beltran-Aguilar E., Mathur M.R., Niederman R. (2021). Pandemic Considerations on Essential Oral Health Care. J. Dent. Res..

[48] Ahmat A., Okoroafor S.C., Kazanga I., Asamani J.A., Millogo J.J.S., Illou M.M.A., Mwinga K., Nyoni J. (2022). The health workforce status in the WHO African Region: Findings of a cross-sectional study. BMJ Global Health.

[49] Nash D.A., Friedman J.W., Mathu-Muju K.R., Robinson P.G., Satur J., Moffat S., Kardos R., Lo E.C., Wong A.H., Jaafar N. (2014). A review of the global literature on dental therapists. Community Dent. Oral Epidemiol..

[50] Pucca G., Gabriel M., De Araujo M., De Almeida F. (2015). Ten years of a National Oral Health Policy in Brazil: Innovation, boldness, and numerous challenges. J. Dent. Res..

[51] Afriyie D.O., Nyoni J., Ahmat A. (2019). The state of strategic plans for the health workforce in Africa. BMJ Glob. Health.

[52] Charlotte S.N., Ahmed A., Zulfa A., Atalay A., Ricardo A., Max B., Alemayehu B., Birke B., Nataliya B., Dixon C. (2022). HeAlth System StrEngThening in four sub-Saharan African countries (ASSET) to achieve high-quality, evidence-informed surgical, maternal and newborn, and primary care: Protocol for pre-implementation phase studies. Glob. Health Action.

[53] Kruk M.E., Gage A.D., Arsenault C., Jordan K., Leslie H.H., Roder-DeWan S., Adeyi O., Barker P., Daelmans B., Doubova S.V. (2018). High-quality health systems in the Sustainable Development Goals era: Time for a revolution. Lancet Glob. Health.

[54] World Health Organization (2022). Global Oral Health Status Report: Towards Universal Health Coverage for Oral Health by 2030.

[55] Lassi Z.S., Cometto G., Huicho L., Bhutta Z.A. (2013). Quality of care provided by mid-level health workers: Systematic review and meta-analysis. Bull. World Health Organ..

[56] World Health Organization, Task shifting: Global recommendations and guidelines. https://apps.who.int/iris/bitstream/handle/10665/43821/9789?sequence=1.

[57] Cahill J. (1995). Making the most of an essential resource. Using skill mix for the benefit of staff and patients. Prof. Nurse.

[58] Buchan J., Dal Poz M.R. (2002). Skill mix in the health care workforce: Reviewing the evidence. Bull. World Health Organ..

